# The draft genomes and investigation of serotype distribution, antimicrobial resistance of group B *Streptococcus* strains isolated from urine in Suzhou, China

**DOI:** 10.1186/s12941-018-0280-y

**Published:** 2018-06-26

**Authors:** Yong Guo, Xiao Deng, Yuan Liang, Liang Zhang, Guo-Ping Zhao, Yan Zhou

**Affiliations:** 1 Institutes of Biology and Medical Sciences, Medical College of Soochow University, Suzhou, 215123 China; 2 State Key Laboratory of Genetic Engineering, School of Life Sciences, Fudan University, Shanghai, 200433 China; 3 Shanghai-MOST Key Laboratory of Health and Disease Genomics, Chinese National Human Genome Center at Shanghai, Shanghai, 201203 China

**Keywords:** Group B *Streptococcus*, Serotype, Antimicrobial susceptibility, Drug resistance gene, Genome

## Abstract

**Background:**

The group B *Streptococcus* (GBS) is a human commensal bacterium, which is capable of causing several infectious diseases in infants, and people with chronic diseases. GBS has been the most common cause of infections in urinary tract of the elders, but relatively few studies reported the urine-isolated GBS and their antimicrobial susceptibilities. Hence, we decided to investigate GBS specially isolated from urine in Suzhou, China.

**Methods:**

27 GBS samples were isolated from urine in Suzhou, China. The PCR and agarose gel electrophoresis were used to identify the serotype distribution. Susceptibility tests were based on MIC test and Kirby–Bauer test. Genome were sequenced via Illumina Hiseq platform and assembled by SPAdes. Genomes of five isolates were sequenced and submitted to NCBI genome database. The sequencing files in fastq format were submitted to NCBI SRA database.

**Results:**

Five serotypes were identified. The resistant rates measured for tetracycline, erythromycin, clindamycin and fluoroquinolones were 74.1, 63.0, 44.4 and 48.1%, respectively. 18.5% of the isolates were nonsusceptible to nitrofurantoin. The resistance to tetracycline was mainly associated with the gene *tetM*. The erythromycin resistance was mainly associated with the genes *ermB* and *mefE*. The genes *ermB* and *lnuB* were the prevalent genes in cMLSB type. No known nitrofurantoin resistance gene was found in nitrofurantoin-nonsusceptible GBS.

**Conclusions:**

Five serotypes were identified in our study. High rates of GBS isolates were resistant to tetracycline, erythromycin, clindamycin and fluoroquinolones. The genes *ermB* and *lnuB* occupied high rates in cMLS_B_ phenotype.

**Electronic supplementary material:**

The online version of this article (10.1186/s12941-018-0280-y) contains supplementary material, which is available to authorized users.

## Background

Group B Streptococcus (GBS, i.e. *Streptococcus agalactiae*) is a gram-positive microbe with bacterial morphology of sphere and oval observed via microscope [1]. Tilapia mossambica and cow are susceptible to GBS, leading to the reduced milk production [2, 3]. GBS can be divided into ten serotypes, Ia, Ib, and II–IX, based on variant capsular polysaccharide. The serotypes Ia, Ib, III and V are the predominant pathogens, primarily liable for human infection [4]. GBS has traditionally been considered as a perinatal pathogen, in relation to certain infectious diseases in pregnant or postpartum women encomapassing endometritis, septicemia, chorioamnionitis and urinary tract infection [5]. Furthermore, GBS has been proved causing severe diseases in infants, such as pneumonia and meningitis [6]. Infectious pattern in infants were separated into two ways: mother-to-child vertically transmitted infection and birth canal transmission [7]. Screening for GBS in pregnant women via swabs taken from the vagina, of which the colonized GBS were mostly harmless, following by antibiotic treatment to prevent the transmission to the babies.

GBS can also cause asymptomatic bacteriuria, cystitis, pyelonephritis, urethritis and urosepsis in urinary tract [8]. In a series of studies, bacteremia and urinary tract infections caused by GBS were the most common diagnoses among people over the age of 70, with 13 of 33 cases (39.4%) [9]. Previous studies indicated that all serotypes of GBS are susceptible to penicillin and have high rates of resistance to tetracycline, clindamycin and erythromycin [10], but little attention was paid to molecular mechanisms underlying those antimicrobial susceptibilities of GBS in urinary tract. A total of 27 GBS samples were isolated from urine in this study to investigate the serotype distribution and antibiotic susceptibilities in Suzhou, China.

## Methods

### Samples collecting and typing

All the GBS samples were isolated from urine between November 2015 and March 2017 in Suzhou Hospital of Traditional Chinese Medicine, Suzhou, China. The isolates were cultured on Blood agar medium at 37 °C with 5% CO_2_ for 24 h. The DNA was extracted via the Ezup Column Bacteria Genomic DNA Purification Kit. The polymerase chain reaction (PCR) and agarose gel electrophoresis were applied (the primer sequences are in Additional file 1: Table S1, the PCR amplification system is in Additional file 1: Table S2 and the procedure of PCR is in Additional file 1: Table S3) to determine the serotype according to the expectation of electrophoresis result (Additional file 1: Table S4).

### Antimicrobial susceptibility test and drug resistance gene PCR

Susceptibility tests to tetracycline, erythromycin, clindamycin, fluoroquinolones, nitrofurantoin, vancomycin, linezolid, quinupristin–dalfopristin and tigecycline were based on the MIC test. Susceptibility to ceftriaxone and penicillin was based on the Kirby–Bauer test recommended by the clinical and laboratory standards institute (CLSI) guidelines [11]. The clindamycin-induced resistance was detected based on D test [12]. The PCR of drug resistance genes was applied to find 12 resistance genes for tetracycline, erythromycin and clindamycin (Additional file 1: Table S5).

### Genome sequencing and bioinformatics analysis

Genomes of four isolates (No.8, No.11, No.17 and No.24) showing multiple resistances and one isolate (No.20) indicating susceptive to all antibiotic were sequenced. The DNA was extracted by QIAGEN DNeasy^®^ Blood & Tissue and sequenced via Illumina Hiseq platform. The genomes were assembled by SPAdes [13] and the k-mer value was set to 127. The scaffolds < 1 kb in length were dropped. The genome sequences were submitted to Antibiotic Resistance Genes Database (ARDB, http://ardb.cbcb.umd.edu/) [14] to query drug resistance genes. The genes were predicted by Prokka (version 1.12).

Two protein sequences of nitrofurantoin-resistant genes, *nsfA* and *nsfB*, were received from The Comprehensive Antibiotic Resistance Database (CARD, https://card.mcmaster.ca/) [15] and then aligned to No.8 genome and No.11 genome sequences via TBLASTN (0.05 e-value) in order to find matching sequences.

The assembled draft genomes of the five isolates were submitted to NCBI genome and SRA database (SAMN08287475, SAMN08287476, SAMN08287477, SAMN08287478 and SAMN0828747). The sequencing files were submitted to NCBI SRA database (SRP128497).

Six complete serotype III genomes were download from NCBI (NZ_CP022537.1/CP022537.1, NZ_CP010874.1/CP010874.1, NZ_CP010875.1/CP010875.1, NZ_CP011327.1/CP011327.1, NZ_CP011329.1/CP011329.1 and NC_004368.1/AL732656.1), respectively. The orthologous list of isolates No.8, No.11 and genomes downloaded from NCBI was built via orthoMCL (v1.4). The orthologous families which contain genes in isolates No.8, No.11 genome and in at most one complete genome downloaded from NCBI, genes in isolates No.8 genome and in at most one complete genome downloaded from NCBI, genes in isolates No.11 and in at most one complete genome downloaded from NCBI, genes in isolates No.8 and No.11 that did not be recorded to orthologous list were extracted by our own PERL script. We also built another orthologous list between isolates No.8 and No.11.

The orthologous list of isolates No.8, No.11, No.17, No.20 and No.24 was built via orthoMCL (v1.4). The orthologous families which contain genes both in isolates No.8 and No.11 genome were also extracted by our own PERL script.

## Results

Group B *Streptococcus* can cause urinary tract infections (UTI). From November 2015 to March 2017 in Suzhou Hospital of Traditional Chinese Medicine, there were 147 individuals had positive urine culture of gram-positive bacteria and 30 (20.4%) of them were positive for GBS. However, few studies reported the GBS in urinary tract. Hence, we presented 27 GBS samples isolated from urine to investigate potential clinical treatment of GBS infections in Suzhou, China.

### Serotypes

Five serotypes were found in 27 samples, which were Ia, Ib, III, V and VI. The proportion and number were 18.5% (5/27), 22.2% (6/27), 29.6% (8/27), 22.2% (6/27) and 7.4% (2/27) respectively (Table 1).

**Table 1:** PCR result of antibiotic resistance genes

Serotype	Sample	*tetM*	*tetO*	*tetK*	*tetL*	*ermA*	*ermB*	*ermC*	*ermM*	*ermTR*	*mefA*	*mefE*	*lnuB*	Resistance summary	D test
III	13	+	−	−	−	−	+	−	−	−	−	−	+	TET, FQNS, cMLS_B_	/
III	18	+	−	−	−	−	−	−	−	−	−	−	+	TET, FQNS, cMLS_B_	/
III	27	+	−	−	−	−	+	−	−	−	−	−	+	TET, FQNS, cMLS_B_	/
III	1	+	−	−	−	−	−	−	−	−	−	+	+	TET, FQNS, cMLS_B_	/
V	26	+	−	−	−	−	+	−	−	+	−	+	+	TET, FQNS, cMLS_B_	/
Ib	8	−	+	−	−	−	+	−	−	−	−	+	+	TET, cMLS_B_	/
Ib	24	+	−	+	+	−	+	−	−	−	−	−	+	TET, cMLS_B_	/
Ia	3	−	+	−	−	−	+	−	−	−	−	−	−	TET, cMLS_B_	/
III	14	+	−	−	−	−	−	−	−	−	−	−	−	TET, FQNS, MS	Negative
V	17	+	−	−	−	−	−	−	−	+	−	+	−	TET, FQNS, iMLS_B_	Positive
V	10	+	−	−	−	−	−	−	−	+	−	+	−	TET, FQNS, iMLS_B_	Positive
Ib	4	−	−	−	−	−	−	−	−	−	−	−	−	FQNS, cMLS_B_	/
Ib	22	−	−	−	−	−	−	−	−	−	−	−	−	FQNS, cMLS_B_	/
Ib	15	−	−	−	−	−	−	−	−	−	−	−	−	CLI, FQNS	/
III	11	+	−	−	−	−	−	−	−	−	−	−	−	TET, iMLS_B_	Positive
Ia	16	+	−	−	−	−	−	−	−	−	−	−	−	TET, MS	Negative
Ia	2	+	−	−	−	−	−	−	−	−	−	−	−	TET, MS	Negative
III	6	+	−	−	−	−	−	−	−	−	−	−	−	TET, MS	Negative
Ia	5	+	−	−	−	−	−	−	−	−	−	−	−	TET, CLI	/
Ib	7	−	−	−	−	−	−	−	−	−	−	−	−	FQNS	/
III	9	+	−	−	−	−	−	−	−	−	−	+	+	FQNS	/
Ia	23	+	−	−	−	−	−	−	−	−	−	−	−	TET	/
V	25	−	+	−	−	−	−	−	−	−	−	+	−	TET	/
V	19	+	−	−	−	−	+	−	−	−	−	−	−	TET	/
V	21	+	−	−	−	−	−	−	−	−	−	−	−	TET	/
VI	12	−	−	−	−	−	−	−	−	−	−	−	−	−	/
VI	20	−	−	−	−	−	−	−	−	−	−	−	−	−	/
Total number		18	3	1	1	0	10	0	0	3	0	9	8		

The order was sorted based on Additional file 1: Table S6. *Tet* genes are resistant to tetracycline; *erm* genes are resistant to clindamycin, erythromycin and streptogramin_b; *mef* genes are resistant to erythromycin; *lnuB* gene is resistant to clindamycin. Erythromycin is a kind of macrolides; clindamycin a kind of lincosamides

*TET* tetracycline, *CLI* clindamycin, *E* erythromycin, *FQNS* fluoroquinolones. *cMLS*_*B*_ constitutive resistance to macrolides, lincosamides, and streptograminB, *iMLS*_*B*_ inducible resistance to macrolides, lincosamides, and streptograminB, *MS* resistance to macrolides and susceptibility to lincosamides

### Resistance of tetracycline, erythromycin and clindamycin

All the 27 samples were susceptible to ceftriaxone, penicillin, vancomycin, linezolid, quinupristin–dalfopristin and tigecycline, while the highest percentage of 74.1% (20/27, Table 1 and Additional file 1: Table S6) samples were resistant to tetracycline. In previous studies, only the PCR primers of genes involved in resistance to tetracycline, erythromycin and clindamycin has been reported. In our PCR test of antibiotic resistance genes, *tetM* was the dominating resistance gene to tetracycline with 90.0% (18/20) occurrence. Other genes resistant to tetracycline were *tetO*, *tetK* and *tetL*, and the rates were 15.0% (3/20), 5.0% (1/20) and 5.0% (1/20) respectively.

The resistant rate measured for erythromycin was 63.0% (17/27). *ErmB*, *mefE* and *ermTR* were found and the rates are 47.1% (8/17), 41.2% (7/17) and 17.6% (3/17) respectively. About 44.4% (12/27) isolates were resistant to clindamycin. *ErmB* 75.0% (9/12)*, lnuB* 58.3% (7/12) *and ermTR* 8.3% (1/12) were found in these isolates. Seven isolates were resistant to erythromycin but susceptible to clindamycin. Three of them were ‘inducible resistance to macrolides, lincosamides and streptograminB, (iMLS_B_) phenotype since they were positive in D test (Additional file 2: Figure S1). Four isolates were ‘resistance to macrolides and susceptibility to lincosamides (MS)’ phenotype. The *erm* (erythromycin ribosome methylase) gene has been considered as the reason of resistance to erythromycin, clindamycin and streptograminB, or the cMLS_B_. There were ten isolates resistant to erythromycin and clindamycin (cMLS_B_) with a high rate of *ermB* (80.0%) and *lnuB* (70.0%) detection. Five cMLS_B_ isolates have both *ermB* and *lnuB* genes. Three cMLS_B_ isolates have *ermB* gene without *lnuB* gene, two isolates have *lnuB* gene without *ermB* gene.

The PCR tests of common drug resistance genes (resistance genes for tetracycline, erythromycin and clindamycin) could explain 74.1% phenotype of drug resistances, except for the seven isolates (No.2, No.5, No.9, No.11, No.14, No.19 and No.25, Table 1 and Additional file 1: Table S6). We sequenced the genome of No.11 isolate and found another known erythromycin resistance gene, *ermT* (Table 3), suggesting that potentially there were other known resistance genes in the remaining six isolates.

### Multiple resistance and detection for novel antibiotic resistance genes

Isolates of No.8, No.11, No.17 and No.24 were nonsusceptible to at least three antibiotics. As the control, No.20 isolate was susceptible to all antibiotics. These five isolates were sequenced. The draft genome sizes were from 2.06 to 2.20 MB, and the scaffold N50 s were from 107,763 to 468,051 bp (Table 2). As expected, none known resistance genes were found in PCR test and draft genome of No.20 isolate.

**Table 2:** Genome information

Strain	Serotype	Total scaffolds number	Total length (bp)	Gap length (N) (bp)	Average length (bp)	Minimum length (bp)	Maximum length (bp)	GC content (%)	N50 (bp)
8	Ib	32	2,200,680	190	68,771	1085	394,159	35.64	183,219
11	III	41	2,107,554	92	51,403	1045	247,508	35.53	107,763
17	V	34	2,106,885	0	61,967	1213	265,957	35.47	117,386
20	VI	11	2,061,455	104	187,405	1064	1,656,169	35.39	1,656,169
24	Ia	20	2,113,859	97	105,692	1036	712,759	35.38	468,051

The No.8 isolate was resistant to tetracycline, erythromycin, clindamycin, and intermediate to nitrofurantoin. Genes *tetO* (tetracycline resistant), *ermB* (erythromycin and clindamycin resistant), *mefE* (erythromycin resistant) and *lnuB* (clindamycin resistant) were found in the PCR test. Genes *tetO* (tetracycline resistant), *mefA* (erythromycin resistant) and *lnuB* (clindamycin resistant) were found in the draft genome. No nitrofurantoin resistance gene was found (Tables 1, 3). No.11 isolate was resistant to tetracycline, erythromycin, and intermediate to nitrofurantoin. Tetracycline resistance gene *tetM* was found in PCR test and in the draft genome, but no erythromycin resistance gene was found in PCR test. An erythromycin and clindamycin resistance gene *ermB* was found in the draft genome. However, no nitrofurantoin resistance gene was found. About 48.1% (13/27, Additional file 1: Table S6) of the isolates were resistant to fluoroquinolones. One of them, No.17 isolate, was resistant to tetracycline, erythromycin and fluoroquinolones. Fluoroquinolones resistance gene was not included in the targets of PCR test, but the *pmrA* was found in the draft genome. The *tetM* (tetracycline resistant) was found in PCR test and in the draft genome. The *ermTR* (erythromycin and clindamycin resistant) and *mefE* (erythromycin resistant) were found in PCR test. The *mefA* (erythromycin resistant) was found in the draft genome. No.24 isolate was resistant to tetracycline, erythromycin and clindamycin. Tetracycline resistance genes *tetM*, *tetK* and *tetL*, erythromycin and clindamycin resistance gene *ermB* and clindamycin resistance gene *lnuB* were found in PCR test. Tetracycline resistance genes *tetM* and *tetL* and erythromycin resistance gene *macB* were found in the draft genome.

**Table 3:** Known antibiotic resistance genes in draft genomes

Sample no.	Resistance of phenotype	Gene name	Resistance of genotype
8	Tetracycline, erythromycin, clindamycin	*lnuB*	Clindamycin
		*mefA*	Erythromycin
		*tetO*	Tetracycline
11	Tetracycline, erythromycin	*ermT*	Clindamycin, erythromycin
		*tetM*	Tetracycline
17	Tetracycline, erythromycin, fluoroquinolones	*tetM*	Tetracycline
		*mefA*	Erythromycin
		*pmrA*	Fluoroquinolone
20	None	none	None
24	Tetracycline, erythromycin, clindamycin	*tetL*	Tetracycline
		*tetM*	Tetracycline
		*macB*	Erythromycin

These genomes were submitted to ARDB database to query known resistance genes

About 18.5% (5/27, Additional file 1: Table S6) of the isolates were intermediate to nitrofurantoin, among which four nitrofurantoin-intermediate isolates were serotype III and the other one was serotype Ib. So far, two nitrofurantoin-resistant genes, *nsfA* and *nsfB*, have been reported [16]. About two-thirds of the length of *nsfB* protein were aligned to all of the five draft genomes by TBLASTN search (E-value threshold 0.05, Additional file 1: Table S7), in which the genome area contained a ‘Ribosomal large subunit pseudouridine synthase B’ f in No.8 isolate, a ‘glyoxal reductase’ in No.11, a “putative NAD(P)H nitroreductase YfkO” in No.17, No.20 and No.24 isolates. So that the phenotype of nitrofurantoin intermediation was not caused by *nsfB* in our cases.

Based on the assumption that the mechanism of the nitrofurantoin intermediation in two isolates was similar, six complete serotype III genomes download from NCBI were included and built orthologous list with genomes of the nitrofurantoin-intermediate samples to search for candidate genes, given that the nitrofurantoin intermediation could not be explained by reported nitrofurantoin-resistant genes. Considering that a detailed information for nitrofurantoin resistance of these complete serotype III genomes is limited, we supposed that at most one of these genomes has a nitrofurantoin-resistant phenotype according to the reference resistance occurrence. Seventeen orthologous contain genes in genomes of No.8, No.11 isolates only or mixed with at most one complete genome downloaded from NCBI. There were 18 genes of isolate No.8 and 17 genes of isolate No.11 in these seventeen orthologous (Additional file 1: Table S8). Most of those genes (16 of 35) were annotated as “hypothetical proteins” since their biological function has still not been clarified. We also identified several genes associated with transposition (6 of 35) and bacteriolysis (2 of 35), which appear to be unessential for the nitrofurantoin intermediation. Therefore, the nitrofurantoin-resistant related genes probably exist in those “hypothetical proteins”. There were 27 genes distributed in the orthologous which contain genes only in isolate No.8 or mixed with at most one NCBI genome. 60 genes were distributed in the orthologous which contain genes only in isolate No.11 or mixed with at most one NCBI genome. Totally 148 genes of No.8 and 53 genes of No.11 were not recorded in this orthologous list. These genes cannot be found in another orthologous list which built between No.8 genome and No.11 genome either, suggesting that these 148 genes in No.8 and 53 genes in No.11 are unique genes of their own.

To further investigate the nitrofurantoin intermediation related genes in isolates No.8 and No.11 genome, the orthologous list of isolates No.8, No.11, No.17, No.20 and No.24 was built, with the other three genomes as the nitrofurantoin-susceptible control. A high proportion of genes were annotated as “hypothetical proteins” (10 of 28, Additional file 1: Table S9), which was consistent with the above-mentioned observation. On the basis of our findings, it can be predicted that there is a higher possibility for the intersection of genes identified in both two orthologous lists to be the candidate genes, which were classified as hypothetical protein, helix-turn-helix domain protein and recombinase. With the range of candidates narrowed down, the four genes annotated as hypothetical proteins (Additional file 1: Table S9) were more likely to be the nitrofurantoin-resistant related genes, providing valuable reference for future experimental study.

## Discussion

Five serotypes were detected in our research, encompassing Ia, Ib, III, V and VI. The serotype III, V and Ib were of the highest proportion. The serotype III GBS was the most common type detected in our isolates, which was in line with studies from other countries [17, 18].

All of the GBS isolates studied in this work were susceptible to penicillin, indicating that penicillin is still a considerable choice for treatment of GBS infection. There was high rate of resistance to tetracycline (74.1%) in the test. However, this rate was lower than that reported in Beijing city (83.9%, isolated from vagina or rectum) [19], Taiwan (97.2%, isolated from urine, genital tract, genital tract, wound or pus, blood and respiratory tract) [20] and North American (89.2%, isolated from vagina or rectum) [17]. Compared to Shanghai, a city close to Suzhou, the rate of resistance to erythromycin in Suzhou (63.0%) is higher than reported in Shanghai (51.9%), however the rate of resistance to clindamycin in Suzhou (44.4%) is lower than that of Shanghai (61.5%) [21]. Note that, 80% of the nitrofurantoin-nonsusceptible GBSs in Suzhou were serotype III isolates, much higher than the 29.6% proportion of serotype III occurrence in the studied samples.

The *tetM* gene was the prevalent resistance gene (18/20) in tetracycline resistance isolates. The other detected tetracycline-nonsusceptible related genes were *tetO*, *tetK* and *tetL*. Besides, *ermB* (8/17) was the prevalent gene related to erythromycin resistance, which had been reported in Korea (isolated from vagina) [22]. It has been reported that the predominant genes in cMLS_B_ were *ermB* in Spain [23, 24] and *ermB* and *ermB*-*mef(A/F*) in Shanghai city [21]. Interestingly, except *ermB*, we found *lnuB* also showed a high proportion (7/10) in cMLS_B_ in Suzhou (70.0%), five isolates have both *ermB* and *lnuB* genes, three isolates have *ermB* gene only and two isolates have *lnuB* gene only. Although this high rate of *lnuB* had not been reported in previous studies.

We suspected that new resistance genes probably exist in the genomes of isolates No.8 and No.11 since the phenotype of nitrofurantoin intermediation in our cases was not caused by *nsfA* and *nsfB*, nitrofurantoin-resistant genes that were recorded in CARD database. If the nitrofurantoin resistance mechanism is similar in the two samples, the 35 genes in the orthologous which contain genes both in the genomes of isolates No.8 and No.11 only or mixed with at most one NCBI genome would be potential candidates for the nitrofurantoin resistance. We also built another orthologous list with the other three genomes of the five isolates as the nitrofurantoin-susceptible control to integrate our finding. There is a greater likelihood for the four genes annotated as hypothetical proteins in the intersection of the two orthologous lists to be the nitrofurantoin-resistant related genes.

## Conclusions

We isolated 27 GBS samples from urine in Suzhou, China. Five serotypes were identified (Ia, Ib, III, V and VI). The serotype III was the primary type. All of the samples were susceptible to ceftriaxone, penicillin, vancomycin, linezolid, quinupristin–dalfopristin and tigecycline. The resistance rates to tetracycline, erythromycin, clindamycin and fluoroquinolones were 74.1, 63.0, 44.4 and 48.1% respectively. The resistance to tetracycline was mainly associated with the gene *tetM* (18/20). The erythromycin resistance was mainly associated with the genes *ermB* (8/17) and *mefE* (7/17). The genes *ermB* (8/10) and *lnuB* (7/10) occupied high rates in cMLS_B_ phenotype. We found five samples were nonsusceptible to nitrofurantoin, and four of them were serotype III, among which two genomes of isolates do not contain any reported nitrofurantoin resistance genes. Five isolates genomes were sequenced and submitted to NCBI genomes database (SAMN08287475, SAMN08287476, SAMN08287477, SAMN08287478 and SAMN0828747) and SRA database (SRP128497).

## Additional files

**Additional file 1: Table S1.** The primers of serotype testing PCR. **Table S2.** The PCR amplification system of cps gene. **Table S3.** The procedure of PCR amplification system for cps gene. **Table S4.** The PCR amplification products of serotype Ia to IX. **Table S5.** The primers of drug resistance genes. **Table S6.** Antimicrobial susceptibility test result. **Table S7.** TBLAST result of nsfB against five draft genomes. **Table S8.** The function and numbers of genes in orthologous which contain genes in genomes of isolates both No.8 and No.11 only or mixed with at most one NCBI genome. **Table** **S9.** The function and numbers of genes in orthologous which contain genes in genomes of isolates No.8, No.11, No.17, No.20 and No.24.

**Additional file 2: Figure S1.** Positive results (iMLSB) in D-test. From left to right are No.10, No.11 and No.17, respectively.

## Abbreviations

GBS: group B *Streptococcus*
UTI: urinary tract infections
cMLSB: constitutive resistance to macrolides, lincosamides, and streptograminB
iMLSB: inducible resistance to macrolides, lincosamides, and streptograminB
MS: resistance to macrolides and susceptibility to lincosamides
CLSI: clinical and laboratory standards institute guidelines
NCBI: National Center for Biotechnology Information
ARDB: Antibiotic Resistance Genes Database
CARD: Comprehensive Antibiotic Resistance Database
PCR: the polymerase chain reaction
MIC: minimal inhibit concentration
