# Supplementary material for: The draft genomes and investigation of serotype distribution, antimicrobial resistance of group B Streptococcus strains isolated from urine in Suzhou, China
Source: Ann Clin Microbiol Antimicrob. 2018 Jun 26;17:28. doi: 10.1186/s12941-018-0280-y (PMC6020191; doi:10.1186/s12941-018-0280-y)
Supplement: Supplementary file 1 — Additional file 1: Table S1. The primers of serotype testing PCR. Table S2. The PCR amplification system of cps gene. Table S3. The procedure of PCR amplification system for cps gene. Table S4. The PCR amplification products of serotype Ia to IX. Table S5. The primers of drug resistance genes. Table S6. Antimicrobial susceptibility test result. Table S7. TBLAST result of nsfB against five draft genomes. Table S8. The function and numbers of genes in orthologous which contain genes in genomes of isolates both No.8 and No.11 only or mixed with at most one NCBI genome. Table S9. The function and numbers of genes in orthologous which contain genes in genomes of isolates No.8, No.11, No.17, No.20 and No.24. [file 12941_2018_280_MOESM1_ESM.docx]

Table S1

The primers of serotype testing PCR

| No. | primer | sequence（5′-3′） |
| --- | --- | --- |
| 1 | cpsI-Ia-6-7-F | GAATTTATAACTTTTGTAGATAGCGATGA |
| 2 | cpsI-6-R | CAATTCTGTCGGACTATCCTGATG |
| 3 | cpsI-7-R | TGTCGCTTCCACACTGAGTGTTGA |
| 4 | cpsL-F | CAATCCTAAGTATTTTCGGTTCATT |
| 5 | cpsL-R | TAGGAACATGTTCATTAACATAGC |
| 6 | cpsG-F | ACATGAACAGCAGTTCAACCGT |
| 7 | CpsG-R | ATGCTCTCCAAACTGTTCTTGT |
| 8 | CpsG-2-3-6-R | TCCATCTACATCTTCAATCCAAGC |
| 9 | CpsN-5-F | ATGCAACCAAGTGATTATCATGTA |
| 10 | CpsN-5-R | CTCTTCACTCTTTAGTGTAGGTAT |
| 11 | CpsJ-8-F | TATTTGGGAGGTAATCAAGAGACA |
| 12 | CpsJ-8-R | GTTTGGAGCATTCAAGATAACTCT |
| 13 | cpsJ-2-4-F | CATTTATTGATTCAGACGATTACATTGA |
| 14 | cpsJ-2-R | CCTCTTTCTCTAAAATATTCCAACC |
| 15 | cpsJ-4-R | CCTCAGGATATTTACGAATTCTGTA |
| 16 | cpsI-7-9-F | CTGTAATTGGAGGAATGTGGATCG |
| 17 | cpsI-9-R | AATCATCTTCATAATTTATCTCCCATT |
| 18 | cpsJ-Ib-F | GCAATTCTTAACAGAATATTCAGTTG |
| 19 | cpsJ-Ib-R | GCGTTTCTTTATCACATACTCTTG |

Table S2

The PCR amplification system of cps gene

| PCR system | volume（µl） |
| --- | --- |
| DNA template | 5 |
| Taq PCR Master Mix | 12.5 |
| primer 1、primer 16 | 0.1 |
| primer 2-15、primer 17-19 | 0.0625 |
| ddH2O | 6.2375 |
| total volume | 25 |

Table S3

The procedure of PCR amplification system for cps gene

|  | temperature（℃） | time（min） | cycle number |
| --- | --- | --- | --- |
| pre-denaturation | 95 | 5 | 1 |
| denaturation 1 | 95 | 1 | 15 |
| annealing 1 | 54 | 1 | 15 |
| extension 1 | 72 | 2 | 15 |
| denaturation 2 | 95 | 1 | 25 |
| annealing 2 | 56 | 1 | 25 |
| extension 2 | 72 | 2 | 25 |
| final extension | 72 | 10 | 1 |

Table S4

The PCR amplification products of serotype Ia to IX

| Ia | Ib | II | III | IV | V | VI | VII | VIII | IX |
| --- | --- | --- | --- | --- | --- | --- | --- | --- | --- |
| 688(cpsL) | 688(cpsL) | 688(cpsL) | 688(cpsL) | 688(cpsL) | 688(cpsL) | 688(cpsL) | 688(cpsL) | 688(cpsL) | 688(cpsL) |
|  | 622(cpsJ) |  |  |  |  |  |  |  |  |
|  |  |  |  |  | 582(cpsN) |  |  |  |  |
|  |  |  |  | 538(cpsJ) |  |  |  |  |  |
|  |  |  |  |  |  | 471(cpsI) |  |  |  |
|  |  | 465(cpsJ) |  |  |  |  |  |  |  |
|  |  |  |  |  |  |  |  | 438(cpsJ) |  |
|  |  |  | 352(cpsG) |  |  | 352(cpsG) |  |  |  |
| 274(cpsG) | 274(cpsG) | 274(cpsG) |  | 274(cpsG) | 274(cpsG) |  | 274(cpsG) |  | 274(cpsG) |
|  |  |  |  |  |  |  |  |  | 229(cpsI) |
|  |  |  |  |  |  |  | 179(cpsI) |  |  |

Table S5

The primers of drug resistance genes

| primer | sequence | Product size (bp) |
| --- | --- | --- |
| *tetM*^F^ | 5 -TTATCAACGGTTTATCAGG-3 | 397 |
| *tetM*^R^ | 5 -CGTATATATGCAAGACG-3 |  |
| *tetO*^F^ | 5 -AACTTAGGCATTCTGGCTCAC-3 | 515 |
| *tetO*^R^ | 5 -TCCCACTGTTCCATATCGTCA-3 |  |
| *tetK*^F^ | 5 -TCCTGGAACCATGAGTGT-3 | 189 |
| *tetK*^R^ | 5 -AGATAATCCGCCCATAAC-3 |  |
| *tetL*^F^ | 5 -TGAACGTCTCATTACCTG-3 | 993 |
| *tetL*^R^ | 5 -ACGAAAGCCCACCTAAAA-3 |  |
| *ermA*^F^ | 5 -TCTAAAAAGCATGTAAAAGAA-3 | 645 |
| *ermA*^R^ | 5 -CTTCGATAGTTTATTAATATTAGT-3 |  |
| *ermB*^F^ | 5 -GAAAAGGTACTCAACCAAATA-3 | 639 |
| *ermB*^R^ | 5 -AGTAACGGTACTTAAATTGTTTAC-3 |  |
| *ermC*^F^ | 5 -TCAAAACATAATATAGATAAA-3 | 642 |
| *ermC*^R^ | 5 -GCTAATATTGTTTAAATCGTCAAT-3 |  |
| *ermM*^F^ | 5 -TCGGCTCAGGAAAAGGG-3 | 658 |
| *ermM*^R^ | 5 -CAAGTTAAGGATGCAGT-3 |  |
| *ermTR*^F^ | 5 -TTGGGTCAGGAAAAGGA-3 | 385 |
| *ermTR*^R^ | 5 -GGGTGAAAATATGCTCG-3 |  |
| *mefA*^F^ | 5 -CGTAGCATTGGAACAGC-3 | 316 |
| *mefA*^R^ | 5 -TGCCGTAGTACAGCCAT-3 |  |
| *mefE*^F^ | 5 -CGTAGCATTGGAACAGC-3 | 513 |
| *mefE*^R^ | 5 -TCGAAGCCCCCTAATCTT-3 |  |
| *lnuB*^F^ | 5 -CCTACCTATTGTTTGTGGAA -3 | 944 |
| *lnuB*^R^ | 5 -ATAACGTTACTCTCCTATTC -3 |  |

Table S6

Antimicrobial susceptibility test result. The order was sorted by the number of antibiotics that GBSs were resistant to.

TET: tetracycline, CLI: clindamycin, E: erythromycin, FQNS: fluoroquinolones, NIT: nitrofurantoin, CRO: ceftriaxone, PEN: penicillin, VAN: vancomycin, LNZ: linezolid, QDA: quinupristin-dalfopristin, TGC: tigecycline.

R: resistant, I: Intermediate, S; sensitive.

cMLS_B_, constitutive resistance to macrolides, lincosamides, and streptograminB; iMLS_B_, inducible resistance to macrolides, lincosamides, and streptograminB; MS, resistance to macrolides and susceptibility to lincosamides.

Erythromycin is a kind of macrolides; clindamycin a kind of lincosamides.

| Serotype | Sample | CLI | E | D-test | TET | FQNS | NIT | CRO | PEN | VAN | LNZ | QDA | TGC | resistance summary |
| --- | --- | --- | --- | --- | --- | --- | --- | --- | --- | --- | --- | --- | --- | --- |
| Ⅲ | 13 | R | R | / | R | R | I | S | S | S | S | S | S | TET, FQNS, cMLS_B_ |
| Ⅲ | 18 | R | R | / | R | R | I | S | S | S | S | S | S | TET, FQNS, cMLS_B_ |
| Ⅲ | 27 | R | R | / | R | R | I | S | S | S | S | S | S | TET, FQNS, cMLS_B_ |
| Ⅲ | 1 | R | R | / | R | R | S | S | S | S | S | S | S | TET, FQNS, cMLS_B_ |
| Ⅴ | 26 | R | R | / | R | R | S | S | S | S | S | S | S | TET, FQNS, cMLS_B_ |
| Ⅰb | 8 | R | R | / | R | S | I | S | S | S | S | S | S | TET, cMLS_B_ |
| Ⅰb | 3 | R | R | / | R | S | S | S | S | S | S | S | S | TET, cMLS_B_ |
| Ⅰa | 24 | R | R | / | R | S | S | S | S | S | S | S | S | TET, cMLS_B_ |
| Ⅲ | 14 | S | R | negative | R | R | S | S | S | S | S | S | S | TET, FQNS, MS |
| Ⅴ | 17 | S | R | positive | R | R | S | S | S | S | S | S | S | TET, FQNS, iMLS_B_ |
| Ⅴ | 10 | S | R | positive | R | R | S | S | S | S | S | S | S | TET, FQNS, iMLS_B_ |
| Ⅰb | 4 | R | R | / | S | R | S | S | S | S | S | S | S | FQNS, cMLS_B_ |
| Ⅰb | 22 | R | R | / | S | R | S | S | S | S | S | S | S | FQNS, cMLS_B_ |
| Ⅰb | 15 | R | I | / | S | R | S | S | S | S | S | S | S | CLI, FQNS |
| Ⅲ | 11 | S | R | positive | R | S | I | S | S | S | S | S | S | TET, iMLS_B_ |
| Ⅰa | 16 | S | R | negative | R | S | S | S | S | S | S | S | S | TET, MS |
| Ⅰa | 2 | S | R | negative | R | S | S | S | S | S | S | S | S | TET, MS |
| Ⅲ | 6 | S | R | negative | R | S | S | S | S | S | S | S | S | TET, MS |
| Ⅰa | 5 | R | S | / | R | S | S | S | S | S | S | S | S | TET, CLI |
| Ⅰb | 7 | S | S | / | S | R | S | S | S | S | S | S | S | FQNS |
| Ⅲ | 9 | S | S | / | S | R | S | S | S | S | S | S | S | FQNS |
| Ⅰa | 23 | S | S | / | R | S | S | S | S | S | S | S | S | TET |
| Ⅴ | 25 | S | S | / | R | S | S | S | S | S | S | S | S | TET |
| Ⅴ | 19 | S | S | / | R | S | S | S | S | S | S | S | S | TET |
| Ⅴ | 21 | S | S | / | R | S | S | S | S | S | S | S | S | TET |
| Ⅵ | 12 | S | S | / | S | S | S | S | S | S | S | S | S | - |
| Ⅵ | 20 | S | S | / | S | S | S | S | S | S | S | S | S | - |
| Resistant number | | 12 | 17 |  | 20 | 13 | 0 | 0 | 0 | 0 | 0 | 0 | 0 |  |

Table S7

TBLAST result of *nsfB* against five draft genomes. “*nsfB* start” shows the start of the aligned region of *nsfB* protein, “*nsfB* end” shows the end of the aligned region of *nsfB* protein.

| sample No. | *nsfB* start (aa) | *nsfB* end (aa) | e-value |
| --- | --- | --- | --- |
| 8 | 8 | 168 | 0.001 |
| 11 | 25 | 168 | 0.003 |
| 17 | 8 | 207 | 0.003 |
| 20 | 8 | 207 | 0.003 |
| 24 | 8 | 207 | 0.003 |

Table S8

The function and numbers of genes in orthologous which contain genes in genomes of isolates both No.8 and No.11 only or mixed with at most one NCBI genome

| sample No. | function | gene number |
| --- | --- | --- |
| 8 | hypothetical protein | 7 |
| 8 | Transposase from transposon Tn916 | 2 |
| 8 | Transposon Tn3 resolvase | 1 |
| 8 | HTH-type transcriptional regulator Xre | 1 |
| 8 | rRNA adenine N-6-methyltransferase | 1 |
| 8 | autolysin | 1 |
| 8 | Helix-turn-helix domain protein | 1 |
| 8 | Bacteriophage peptidoglycan hydrolase | 1 |
| 8 | Modification methylase HhaI | 1 |
| 8 | ORF6C domain protein | 1 |
| 8 | Transposon gamma-delta resolvase | 1 |
| 11 | hypothetical protein | 9 |
| 11 | Helix-turn-helix domain protein | 1 |
| 11 | rRNA adenine N-6-methyltransferase | 1 |
| 11 | HTH-type transcriptional regulator Xre | 1 |
| 11 | Mannosyl-glycoprotein endo-beta-N-acetylglucosaminidase | 1 |
| 11 | Transposase from transposon Tn916 | 1 |
| 11 | membrane-bound lytic murein transglycosylase D | 1 |
| 11 | Transposon Tn3 resolvase | 1 |
| 11 | Recombinase | 1 |

Table S9

The function and numbers of genes in orthologous which contain genes in genomes of isolates No8, No11, No.17, No.20 and No.24

| sample No. | function | gene number |
| --- | --- | --- |
| 8 | hypothetical protein^a^ | 4 |
| 8 | Transposase IS66 family protein | 2 |
| 8 | Group II intron-encoded protein LtrA | 2 |
| 8 | FRG domain protein | 2 |
| 8 | Transposon gamma-delta resolvase | 1 |
| 8 | Transposon Tn3 resolvase^c^ | 1 |
| 8 | Helix-turn-helix domain protein | 1 |
| 8 | Replication initiation factor | 1 |
| 8 | Tyrosine recombinase XerC | 1 |
| 11 | hypothetical protein | 6 |
| 11 | Recombinase | 1 |
| 11 | Transposon Tn3 resolvase | 1 |
| 11 | Helix-turn-helix domain protein | 1 |
| 11 | Replication initiation factor | 1 |
| 11 | Putative prophage phiRv2 integrase | 1 |
| 11 | Transcriptional repressor SdpR | 1 |
| 11 | Arsenical resistance operon repressor | 1 |

^a^ The function of genes identified in both two orthologous lists are colored in red
