# Supplementary figures and images for: The draft genomes and investigation of serotype distribution, antimicrobial resistance of group B Streptococcus strains isolated from urine in Suzhou, China
Source: Ann Clin Microbiol Antimicrob. 2018 Jun 26;17:28. doi: 10.1186/s12941-018-0280-y (PMC6020191; doi:10.1186/s12941-018-0280-y)

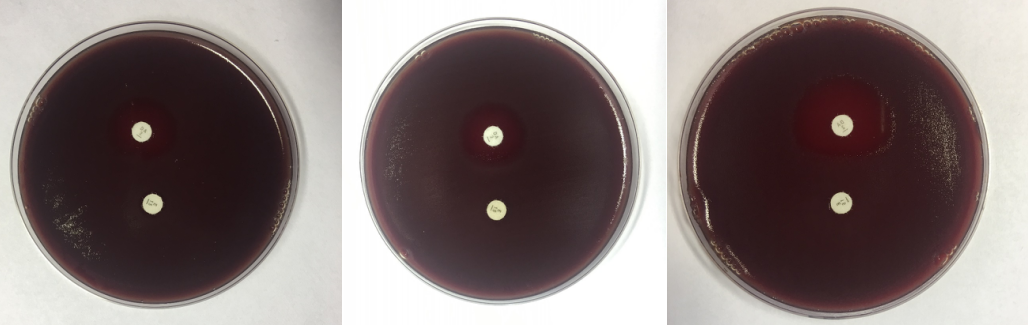

Supplement: Supplementary file 2 — Additional file 2: Figure S1. Positive results (iMLSB) in D-test. From left to right are No.10, No.11 and No.17, respectively. [file 12941_2018_280_MOESM2_ESM.png]
